# Vitamin D supplementation in later life: a systematic review of efficacy and safety in movement disorders

**DOI:** 10.3389/fnagi.2024.1333217

**Published:** 2024-01-26

**Authors:** Carl N. Homann, Barbara Homann, Gerd Ivanic, Tadea Urbanic-Purkart

**Affiliations:** ^1^Department of Neurology, Medical University Graz, Graz, Austria; ^2^St. Elizabeth University of Health and Social Work, Bratislava, Slovakia; ^3^Institute of Human Movement Science, Sport and Health, Graz, Austria; ^4^Institute for Orthopedic and Cardiological Rehabilitation, Privatklinik Ragnitz, Graz, Austria

**Keywords:** vitamin D, supplementation, movement disorders, aging, efficacy, safety, systematic survey, randomized controlled trials

## Abstract

**Introduction:**

Nutrition plays a pivotal role in the multidisciplinary approach to rehabilitating middle to old-aged patients with neurological diseases including movement disorders (MDs). Despite the prevalence of vitamin D deficiency in many patients with MDs, data supporting supplementation’s effectiveness and safety is sparse and conflicting, therefore, our explicit objective was to provide an all-encompassing review of the subject.

**Methods:**

A comprehensive search of PubMed, Embase, and other scientific databases was conducted up to November 1 2023. The searches included RCTs in all languages with human participants aged 35 and above and not meeting these requirements led to exclusion.

**Results:**

Four studies on Parkinson’s disease (PD) and one on restless legs syndrome (RLS) including 369 MD patients, however, none in a rehabilitation context, were found. Although three of the four PD studies showed better outcomes, such as decreasing levodopa-induced dyskinesia or enhancing physical performance in some or all domains, the RLS study did not identify symptom improvement. The one serious adverse effect observed, cerebral infarction, aroused safety concerns, however its relationship to vitamin D consumption is questionable. Structurally the studies can be characterized by large variations in patient populations, in primary outcomes, and disease severity, but typically a relatively short duration of therapy in most cases. With other limitations such as the small number of studies, major trial design heterogeneity, limited sample sizes, and a greatly variable Cochrane risk of bias (RoB) evaluation, only a qualitative synthesis was feasible.

**Discussion:**

Two main implications can be inferred from these results, which we interpret as cautiously promising but overall insufficient for firm recommendations. First, there is an urgent need for more research on the role of vitamin D in MDs in the middle- to older-aged population, particularly during rehabilitation. Second, given the benefits of vitamin D supplementation for those who are deficient, we recommend routine screening and supplementation for MD patients.

## 1 Introduction

MDs encompass a wide spectrum of conditions, including Parkinson’s disease (PD), tremor, restless legs syndrome (RLS), chorea, athetosis, dystonia, ballism, myoclonus, tics, stereotypies, and ataxia. The impact of these MDs is particularly pronounced among the middle-aged and elderly population, affecting approximately 28% of individuals (Wenning et al., 2005). Prevalence rates, however, vary among distinct population groups and specific MD entities. For instance, PD, RLS, tremor, and stereotypies tend to manifest with increasing frequency with advancing age (Yeh et al., 2012; Dionigi, 2015; Orozco et al., 2020; Song et al., 2021). Moreover, MD subtypes like dystonia and chorea exhibit age as a contributory factor influencing onset and severity (Müller et al., 2002; Kay et al., 2017).

It is important to recognize that the impact of MD symptoms is amplified among elderly patients due to the increased complexity of their treatment. Multiple comorbidities, polypharmacy, and age-related changes in brain physiology present significant challenges. Surprisingly, despite the substantial burden experienced by 60% of aging individuals, only 7% receive adequate treatment, revealing a critical healthcare disparity (Wenning et al., 2005). Even for those who are well cared for, the reality is that treatment for MDs is primarily symptomatic and has limitations, particularly in older people. Medications can lose their effectiveness or have disabling side effects, and surgery is reserved for a select set of people. Elderly people are more susceptible to surgical risks and may have other health issues that impair their ability to tolerate certain medications.

Even after decades of concerted efforts, the treatment of MDs remains challenging, especially for the middle-aged and elderly. The limitations of standard medical and surgical treatment options, together with the changing demographics of an increasing number of older people with movement disorders (MD), have led to a steady increase in the importance and prevalence of non-pharmacological exercise (Steiger and Homann, 2019; da Costa et al., 2023) and nutrition-based interventions for MD in aging (Lister, 2020). Within this framework, Vitamin D supplementation, has emerged as a particularly promising approach for alleviating the complexities faced by treating aging individuals with MDs This strategy is supported scientifically by the discovery that brain regions associated with MDs, such as the substantia nigra, subcortical gray nuclei, and thalamus, contain Vitamin D receptors (VDRs). In these domains, vitamin D affects neuronal maturation, differentiation, and neurotransmitter synthesis, encompassing dopamine and acetylcholine, which are essential for the onset and management of MDs (Homann, 2021). Furthermore, vitamin D’s interaction with VDRs in skeletal muscle cells regulates muscle strength, balance, and coordination. Decreased VDR activity is linked to oxidative stress in skeletal muscle, which may have an impact on mitochondrial performance and lead to muscle atrophy (Dzik and Kaczor, 2019). In line with this theory, it has been consistently demonstrated that supplemental vitamin D has regularly been shown to increase muscular strength and balance in aging people (Muir and Montero-Odasso, 2011). Thus, by supporting muscle integrity, optimal vitamin D levels may also help with certain motor symptoms associated with MDs in aging individuals. Vitamin D is also known to improve mood and cognition in the elderly (Alavi et al., 2019; Jia et al., 2019). These motor and non-motor symptoms are major causes of impairment in older individuals with Parkinson’s disease and other MDs, and they are thus prime therapy targets for rehabilitation programs.

To maximize the benefits of rehabilitation for older patients with MD, it is essential that they are in stable physical and mental health and have a low risk of adverse events during participation. Vitamin D may have the potential to contribute to favorable rehabilitation conditions and positively influence primary treatment goals. However, while there is a wealth of evidence in the literature linking various MDs and low vitamin D levels (Homann, 2021), there is not nearly as much information available about vitamin D supplementation. This systematic review aims to investigate whether vitamin D, known for its multiple beneficial effects on brain pathophysiology (Homann, 2021), can positively influence symptoms in older patients with MDs. To do this, we critically reviewed the existing literature for randomized controlled trials (RCTs) on the benefits and limitations of vitamin D supplementation in this specific population. We also searched for RCTs that specifically investigated symptom improvement in the context of rehabilitation. We hypothesized that vitamin D, when combined with rehabilitation, would enhance improvements in key symptoms of MDs and aimed to establish a knowledge base for future research and practical therapeutic applications.

## 2 Materials and methods

### 2.1 Strategy for conducting literature search

Our objective was to conduct a comprehensive systematic literature search to identify relevant studies published before November 1, 2023 that explore the relationship between vitamin D and MDs in middle-aged and older adults. To achieve this, we selected PubMed, EMBASE and Cochrane Library, as our primary databases due to their extensive coverage of medical literature and relevance to our research objectives. In addition, we extended our search to other scientific databases, including Google Scholar, and Elisit.org. Due to the vast number of results produced by Google Scholar and Elisit.org, we initially limited our search for each MD to 10 results and examined the abstracts. If the tenth result was relevant, we reviewed an additional 10 abstracts, continuing until the tenth result no longer met our criteria.

We employed a precise and inclusive search strategy by selecting appropriate MeSH or Emtree terms and text words. These terms were chosen to ensure that we cover all relevant MDs, including PD, tremor, RLS, chorea, athetosis, dystonia, ballism, myoclonus, tic disorders, stereotypies, and ataxia. To obtain relevant articles we then cross-referenced these terms with “vitamin D,” which also included the terms “Cholecalciferol,” „Ergocalciferol,” “Calcidiol,” “Calcitriol,” “25 (OH)D,” and “1, 25(OH)2D.” Lastly, we linked the terms for the specific MDs and Vitamin D with the term “rehabilitation.” This approach ensures transparency and facilitates replication.

To maximize information, we did not limit our search to English-language articles. In cases where articles were not in English but in one of the major international languages, including Arabic, French, German, Italian, Mandarin, Portuguese, Spanish and Russian, we had proficient colleagues who were native speakers to aid with translation. However, there was only one paper in Spanish and one in French that were considered, but finally not included.

Furthermore, we manually reviewed the bibliographies of identified publications, browsing reference lists for cited references, to ensure maximum literature coverage.

### 2.2 Inclusion and exclusion criteria

#### 2.2.1 Inclusion criteria

The inclusion criteria for this review were meticulously designed to ensure the relevance and quality of the studies included. These criteria encompassed the following:

- Participants aged 35 years and older: While the standard age for older adults is 65+, in some studies even 50+, we decided to lower the age limit to 35 due to the limited number of articles meeting the stricter age criterion. This adjustment aims to include valuable studies with participants below the conventional threshold.
- Specified study design involving human participants: Emphasizing the need for human-based research to address our research questions.
- Randomized controlled clinical trials: to ensure quality data.
- a direct relationship between vitamin D and typical MD symptoms: To maintain relevance to our research objectives.

#### 2.2.2 Exclusion criteria

- Exclusion criteria were applied to maintain the rigor and precision of our study. We excluded articles if they failed to meet our inclusion criteria. This means that studies were not eligible when including:
- Participants, being children or young adults aged 18 to 35 years.
- Non-human participants.
- Designs other than randomized controlled clinical trials.
- Objectives with either no or only indirect association with our research topic.

For example, an RCT on the effect of vitamin D on bone mineralization in PD patients would not be considered eligible, but on balance and falls in PD patients would.

We also excluded studies if they were identified as duplicate publications. Duplicate identification was performed by meticulously comparing relevant data such as authors, study design, study location, number of participants, and participant recruitment. This, at times, required full-text reading.

#### 2.2.3 Doubtful cases

Two authors independently reviewed abstracts and full texts. In cases of disagreement, final decisions on inclusion or exclusion were made by a third author.

To maintain transparency and impartiality, cases with unclear information prompted email contact with the respective article authors.

### 2.3 Risk of bias assessment

In our review, we employed the Cochrane Risk of Bias (RoB 1) tool to assess potential bias in the included studies. The RoB 1 tool was chosen due to its comprehensive approach to bias assessment, which includes domains such as bias caused by randomization, deviations from the intended interventions, missing outcome data, bias in measuring outcomes, and bias in selecting the results reported. Thereby it helps enhancing the reliability and validity of our findings and promotes methodological transparency and replicability.

## 3 Results

### 3.1 Studies retrieved and characteristics

**Figure 1:**
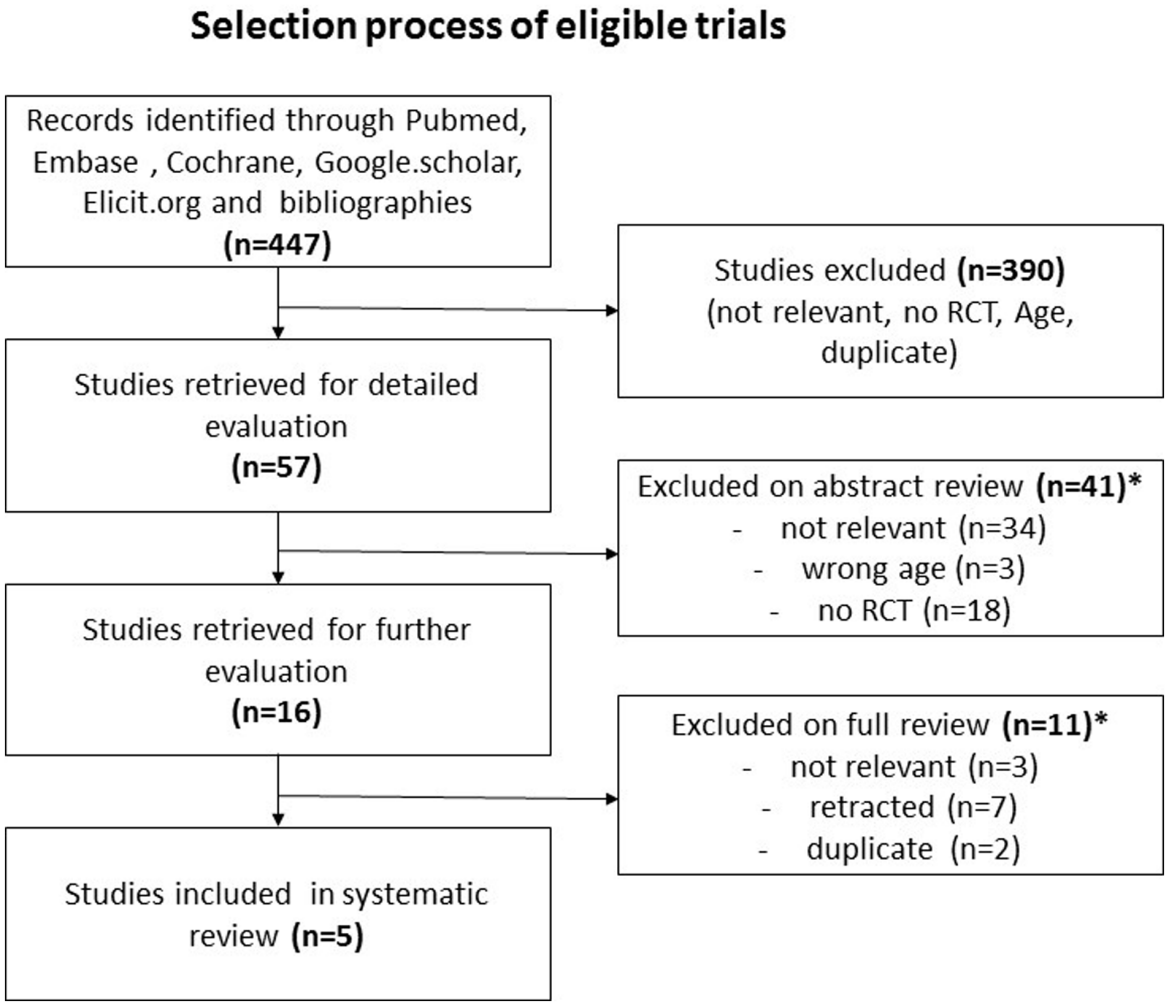
The figure depicts the literature review process: initially, 447 entries were found. After screening and abstract assessment, 57 articles remained. Post full-text review, 5 randomized clinical trials were selected. These trials involved 369 Movement Disorder (MD) patients, with 334 having Parkinson's disease and 35 having restless legs syndrome. *A study can be excluded for more than just one reason.

We discovered 447 entries throughout our extensive search of the literature (Figure 1). An initial screening procedure eliminated 390 articles because they did not fulfill our severe criteria for irrelevance, lack of required study design, differences in age constraints, or duplicate publishing, leaving us with 57. A detailed abstract assessment of these papers resulted in the omission of 41 more studies. Most were unrelated to our research aims (*n* = 34), while others fell short owing to age restrictions (*n* = 3) or a lack of being RCTs (*n* = 18). It should be noted that certain research was rejected for more than one reason. The remaining final selection of 16 papers proceeded through a rigorous full-text review procedure, removing 11 more for reasons such as not being related to the research purpose (*n* = 3), being retracted for fraudulent grounds (*n* = 7), or being duplicates (*n* = 2). As a result, we are left with five randomized clinical trials, four on PD and one on RLS, with a total of 369 MD patients participating, 334 of whom had PD and 35 of whom had RLS (Table 1) For other MD patients, such as individuals suffering from tremors, athetosis, dystonia, ballism, myoclonus, tics, stereotypies, ataxia and chorea including Huntington’s disease, our search did not yield any eligible articles investigating the effects of vitamin D supplementation within the context of randomized controlled trials (RCTs).

**Table 1:** Characteristics and outcome of included randomized clinical trials.

Author	Region	Detailed study design	Pts (*n*)	Age	Intervention	Duration	Outcomes measured	Outcome	Adverse events
Parkinson’s disease									
Bytowska et al. (2023)	Poland	Randomized controlled trial	50	65.6 ± 7.7	VitD3, BMI based: BMI < 25: 4000 IU/d; BMI 25–30: 5000 IU/d; BMI > 30: 6000 IU/d combined with DBS	12 wks	•Physical Performance (TUG, 6 MWT, 10 MWT) •Inflammation Status (CRP) •Serum 25(OH)D3 Concentration •VitD Metabolites	•Increase of VitD concentration • Better functional performance • Trend to reduced inflammation	No information provided
Hiller et al. (2018)	United States	pilot randomized, double blind intervention trial	51	66.6 ± 8.1	VitD3 (10,000 IU/d) plus calcium carbonate, placebo plus 1,000 mg calcium carbonate	16 wks	•Balance (Sensory Organization Test - posturography) •Gait (iMOBILITY) •Strength (Biodex) •Falls (diary, interview) •Cognition (MoCa) •Mood •PD Severity (UPDRS III) •Quality Of Life (NHP, PDQ-39)	•No overall balance improvement. •Potentially improves balance in younger PD patients	High-dose VitD suppl. Safe in PD Detailed analyses: • No serious AEs • No hypercalcemia associated AEs. • Minor AEs: no significant differences between VitD and PL
Habibi et al. (2018)	Iran	randomized, double blind, placebo controlled trial	120	49.9 ± 12.4	VitD3 (1,000 IU/d)	3 mo	•Dyskinesia - duration (per d by h) - severity (UPDRS IV) • UPDRS (Total)	VitD has no effects on improvement of levodopa induced dyskinesia in PD.	No information provided
Suzuki et al. (2013)	Japan	Randomized, double-blind, placebo-controlled trial	114 = VitD 56; PL 58	71.9 ± 6.9	VitD3 (1,200 IU/d)	12 mo	•HY •UPDRS •FokI genotypes	•VitD3 suppl. Stabilizes PD symptoms •FokI TT or CT genotypes have positive effect	• No obvious AEs associated with hypercalcemia • one serious clinical adverse event in the form of cerebral infarction
Restless legs syndrome									
Wali et al. (2019)	KSA	Randomized, placebo controlled trial	22 = VitD 12+ PL 10	42.0 ± 5.1	Vitamin D supplements (50,000 IU/w caplets), placebo	12 wks	• RLSSS (baseline to week 12). •Serum VitD Levels •Bone Profiles	VitD suppl. Does not improve RLS symptoms (RLSSS).	Weekly check for AEs, no info on findings

AEs, adverse events; Biodex, computerized dynamometer for muscle strength; BMI, body mass index: CRP, C-reactive protein; DBS, Deep Brain Stimulation; dysk, dyskinesia; FokI, *Flavobacterium okeanokoites* independent polymorphic site in the VDR gene; HY, Hoehn and Yahr rating scale for Parkinson’s disease; IMOBILITY, device to examine gait during a timed up and go test; IU, international units; KSA, kingdom of Saudi Arabia; mo, months; NHP, MoCa, montreal cognitive assessment test for Dementia; Nottingham health profile; 6 MWT, 6 min walk test; 10 MWT, 10 m walk test; PD, Parkinson’s disease; PDQ-39, the Parkinson’s disease questionnaire; PL, Placebo group; RLS, restless legs syndrome; RLSSS, restless legs syndrome severity score; SOT, sensory organization test - computerized posturography balance measures; TUG, time up and go; UPDRS, unified Parkinson’S disease rating scale; VitD, vitamin D; suppl., supplementation; wks, weeks.

### 3.2 Effect of vitamin D supplementation on movement disorder symptoms

#### 3.2.1 Effect of vitamin D supplementation on Parkinson’s disease

In a 2023 research by Bytowska et al. (2023) 29 PD patients receiving Deep Brain Stimulation (DBS) treatment at a neurosurgical university clinic were subjected to a 12-week Body Mass Index (BMI)-based Vitamin D3 supplementation. The results indicated a remarkable increase in serum 25(OH)D3 concentration (*p* < 0.0006) within the Vitamin D group, along with notable improvements in physical performance, particularly in the Time Up and Go (TUG) (*p* < 0.005) and the six-minute walking (6 MWT) (*p* < 0.05) tests. While the study showed a trend toward improved inflammation status in the Vitamin D group, the sample size might have limited the statistical power of this observation.

A 2018 study by Hiller et al. (2018) focused on the impact of high-dose Vitamin D on balance in 51 PD patients. The study found no substantial differences in balance between the treatment group receiving Vitamin D and calcium carbonate and the placebo group receiving placebo and calcium carbonate. However, a *post hoc* analysis revealed a significant improvement in digitally assessed balance scores (*p* = 0.012) in the younger (ages 52–66), but not in the older (ages 67–86) participant subgroups, suggesting a positive effect on younger patients.

In contrast, a 2018 pilot study by Habibi et al. (2018) explored the effect of lower middle-range Vitamin D regimes on levodopa-induced dyskinesia in 120 PD patients from a tertiary care neurology clinic. After 3 months, the study revealed no significant differences between the Vitamin D and placebo groups in terms of the general PD severity scale (UPDRS-Total), dyskinesia duration (in hours per day), and dyskinesia severity (UPDRS IV).

In 2013, Suzuki et al. (2013) conducted a 12-month study involving 114 participants recruited from 137 consecutive PD patients from a tertiary care setting. The results showed that Vitamin D3 supplementation significantly prevented the deterioration of the Hoehn and Yahr stage in PD patients (*p* < 0.05). This effect was particularly pronounced in patients with specific VDR genotypes (TT or CT).

#### 3.2.2 Effect of vitamin D supplementation on restless legs syndrome

Wali et al. (2019) conducted a 12-week randomized controlled experiment in which 35 RLS patients received Vitamin D in the middle dosage range (50,000 IU caplets/week) or a placebo. To be eligible, potential participants had to go through a rigorous vetting procedure. Initially, 2,600 school personnel were encouraged to complete a symptom survey before being recruited and subjected to more rigorous interviews and assessments. Finally, the 18 RLS patients assigned to vitamin D and the 17 assigned to placebo, were assessed for RLS severity using the International Restless Legs Syndrome Study Group rating scale, and no significant intragroup score differences were identified at the end of the experiment.

### 3.3 Safety of vitamin D supplementation in movement disorders

A total of 369 patients, 354 with PD and 35 with RLS, received study medication, potentially risking the occurrence of adverse events. Among them, 183 were prescribed vitamin D supplementation, while 186 received a placebo.

#### 3.3.1 Safety of vitamin D supplementation in Parkinson’s disease

Among the four PD studies, the description of clinical adverse events varied in detail (Table 2).

**Table 2:** Depiction of study characteristics regarding dropout rates and adverse reaction to vitamin D supplementation and placebo.

Study			Participants				Adverse events				
Author	Duration	VitD dosage	Allocated	Dropout			Clinical causes (VitD: placebo) (% of allocated)				Lab**
			Total (*n*)	VitD [*n* (%)]	Placebo [*n* (%)]	Total [*n* (%)]	Medical Minor	Medical serious	Organi-zational*	Not specified	Results
Parkinson disease											
Bytowska et al. (2023)	12 wks	middle	42	8 (38%)	5 (24%)	13 (31%)			10%: 24%	29%: 0%	
Hiller et al. (2018)	16 wks	high	58	1 (4%)	6 (20%)	7 (17%)	43%: 67%	4%: 0%			All neg
Habibi et al. (2018)	3 mo	low	120	0 (0%)	0	0 (0%)	–	–	–	–	–
Suzuki et al. (2013)	12 mo	low	114	7 (13%)	3 (5%)	10 (9%)			7%: 2%	2%: 0%	All neg
			334	16 (10%)	14 (8%)	23 (7%)					
Restless legs syndrome											
Wali et al. (2019)	12 wks	middle	35	8 (44%)	5 (29%)	13 (37%)	11%: 18%			11%: 29%	
Total			369	24 (13%)	19 (10%)	36 (12%)	n.a.	n.a.	n.a.	n.a.	n.a.

*Organizational adverse events: nonmedical causes for having to discontinue the participation, the most frequent one was the inability to find transport during the COVID pandemic.

**Laboratory screening for abnormal levels of ionized calcium, phosphate, or creatinine, being all in normal recommended limits (all negative).

mo, months; neg, negative; VitD, vitamin D; wks, weeks.

In their comprehensive 16-week high-dose RCT, Hiller et al. (2018) included 28 PD patients receiving vitamin D and 30 receiving a placebo, no serious adverse events were observed. Interestingly, minor adverse events were more frequent in the placebo group (*n* = 20) than in the vitamin D group (*n* = 12) (*p* = 0.32). These minor events included gastrointestinal symptoms, muscle complaints, respiratory symptoms, and other inconsequential difficulties. Serum monitoring in this trial revealed no concerning values, such as abnormal levels of ionized calcium, phosphate, or creatinine, suggesting that this form of vitamin D intervention was safe.

The 12-month middle-range supplementation study by Suzuki et al. (2013), with 56 PD patients allocated to vitamin D and 58 to placebo, coming short of providing a similarly detailed account on all including minor occurrences, nevertheless reported on one serious clinical adverse event in the form of cerebral infarction in the vitamin D3 group (Suzuki et al., 2013). Another withdrawal of consent occurred in the treatment group without a specified reason. Additionally, four patients in the vitamin D group were non-compliant compared to one in the placebo group and were excluded by the investigators. Given the small study population, these differences did not reach statistical significance. Serum safety monitoring meticulously carried out in this trial did not reveal any obvious adverse signs associated with hypercalcemia.

Bytowska et al. (2023) did not disclose specific adverse event counts in their 12-week middle-range intervention study, which included 21 individuals in the treatment arm and 21 in the placebo arm, but did explain dropout causes. Six occurrences were linked to unexplained personal reasons relating to the continuing COVID scenario, and two to organizational issues such as transportation. Five individuals in the placebo arm also dropped out due to transportation issues during these trying times. Thus, the study did not observe any clinical adverse events related to vitamin D supplementation leading to withdrawal.

Unfortunately, Habibi et al. (2018) did not provide information on adverse events or reasons for participant withdrawal in their study, leaving this issue unanswered.

#### 3.3.2 Safety of vitamin D supplementation in restless legs syndrome

During their 12-week randomized controlled trial for RLS symptoms, Wali et al. (2019) conducted serial assessments for adverse events in 18 patients assigned to take vitamin D and 17 patients on placebo. Blood tests, including total vitamin D levels and bone profiles, were performed every 4 weeks. The publication did not explicitly report adverse clinical or laboratory findings. However, dropout rates and their causes were described. Over the study duration, 13 participants dropped out, with eight in the treatment group. Six patients withdrew consent within the first 4 weeks, including two from the Vitamin D group. One of them cited abdominal pain as the reason. In the subsequent 4 weeks, four additional participants reported side effects as withdrawal reasons: one from the Vitamin D group mentioned worsening RLS symptoms, and three from the placebo group claimed to have experienced fatigue or dizziness.

### 3.4 Bias assessment

The Cochrane Risk-of-Bias Tool (RoB 1) indicated that the included RCTs had a mainly low risk in the majority of the areas (Table 3). The exception was the use of subjective questionnaires as the only method of outcome measurement in two trials, one on PD (Habibi et al., 2018) and one on RLS (Wali et al., 2019), which must be categorized as high risk.

**Table 3:** The risk of bias assessment for studies included to a systematic review, conducted using the Cochrane risk-of-bias tool for randomized trials (RoB 1).

Study	Domain 1	Domain 2	Domain 3	Domain 4	Domain 5	Overall Bias
Bytowska et al. (2023)			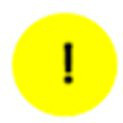			
Hiller et al. (2018)						
Habibi et al. (2018)	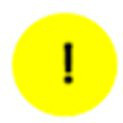		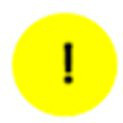	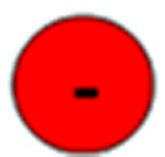		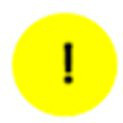
Suzuki et al. (2013)				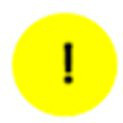		
Wali et al. (2019)				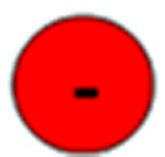		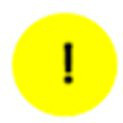

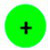
—Low risk of bias; 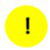
—Some concerns associated with risk of bias; 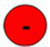
—High risk of bias; Domains: 1—arising from the randomization process; 2—deviations from the intended interventions; 3—missing outcome data; 4—measurement of the outcome; 5—selection of the reported result.

## 4 Discussion

To our knowledge, this is the first systematic review that concentrates on RCTs assessing vitamin D supplementation’s effectiveness and safety throughout the range of MDs in an middle-aged and elderly population. The analysis incorporated five studies, which entailed a total of 369 participants, yielding mixed results. Some studies showed symptom improvement for PD by enhancing physical performance (Bytowska et al., 2023) or alleviating levodopa-induced dyskinesia (Suzuki et al., 2013), while others did not (Hiller et al., 2018) or only partially (Habibi et al., 2018). In the same way, vitamin D had no impact on relieving RLS symptoms (Wali et al., 2019). Although MDs are common in middle-aged and elderly individuals, no RCTs with eligible efficacy trials on additional MDs such as Huntington’s disease, essential tremor, dystonia, or ataxia were discovered. It is obvious that dependable scientific evidence is lacking. Safety monitoring was carried out in two studies, one of which was on PD (Hiller et al., 2018) and one on RLS (Wali et al., 2019), with the detection of a single serious adverse event (Hiller et al., 2018). Limited and only partially positive evidence currently prevents us from conclusively verifying our hypothesis that vitamin D can effectively alleviate symptoms across MDs and be safe. Without RCTs, it is also not possible to make a definitive conclusion about the impact of combining vitamin D with neurorehabilitation.

### 4.1 Discrepancies in efficacy data

The variations in study outcomes can be attributed to several factors and limitations, including those mentioned by the authors in the included papers. Limitations of the studies include a small sample size (ranging from 18 to 60 participants in the treatment groups), significant differences in dosages (ranging from 1,000 IU to 10,000 IU), incomplete treatment adherence (with up to 37% dropout rates), and limited sensitivity of the outcome measures (such as UPDRs Total or Hoehn and Yahr scores). Variations in patient populations, including those recruited from the general public, specialized MD clinics, or specialized DBS clinics, as well as differences in primary outcomes such as dyskinesias, balance and falls, and disease severity, may impact the detection of potential effects of Vitamin D on different subtypes of MDs. Additionally, the relatively short duration of therapy (except for one study, they did not exceed 4 months) and the lack of more severe cases in the studies were cited as potential reasons for these varying results (Suzuki et al., 2013; Wali et al., 2019; Bytowska et al., 2023).

### 4.2 Safety of vitamin D supplementation

The coverage of safety issues in the five included studies was diverse ranging from detailed comprehensive listings (Hiller et al., 2018) to incomplete descriptions (Suzuki et al., 2013), to no accounts at all (Habibi et al., 2018).

Except for one serious adverse event in the form of a cerebral infarct (Hiller et al., 2018), only common general minor events were reported with no significant over-representation of the treatment arm. As expected, gastrointestinal complaints were among the most frequent, in individual cases leading to withdrawals, yet, these also did not happen significantly higher in the supplementation group. Based on the limited number of cases and the occasionally lacking details in the representation, it also appeared that there were no significant differences between higher and lower treatment regimes in the frequency of unwanted events.

High-dose regimens are generally thought to lead to a higher risk of serious events (Bjelakovic et al., 2014). Indeed, the only serious event occurred in the RCT by Hiller et al. (2018), which applied high doses. However, the extensive literature on this topic does not seem to link neither Vitamin D nor the higher dosage regime to cerebral infarction as in this instance. While researchers consider vitamin D deficiency to be associated with ischemic stroke risk, severity, and prognosis (Alharbi et al., 2022), vitamin D3 supplementation, on the other hand, appears to prevent and improve the neurological and psychiatric effects of ischemic stroke (Lasoń et al., 2022).

One serious, but admittedly extremely rare adverse event (Spiller et al., 2016), typically linked to very high doses of vitamin D intake (>10,000 IU/day) is vitamin toxicity (Asif and Farooq, 2023). The most common symptoms are confusion, apathy, vomiting, abdominal pain, excessive urination, thirst, and dehydration. If not treated in time, more serious conditions, such as peptic ulcers, pancreatitis, hypertension, severe cardiac arrhythmias, renal failure, and coma, even death can occur (Asif and Farooq, 2023). In most cases however, despite being rare, outcome is also good, with symptoms subsiding within a few month on adequate treatment (Asif and Farooq, 2023). Hypercalcemia is an essential laboratory sign. Two of our studies mentioned laboratory monitoring, an important preventative step in high-dose interventions, but found no positive results.

Another much-discussed high-dose side effect, particularly in older women, is the occurrence of balance problems resulting in falls and fractures as described by Sanders et al., (2010). It is worth noting that these are already common problems in the elderly with neurological diseases (Homann et al., 2013) and particularly with MDs (Homann, 2021). Sanders et al.’s findings, along with those of other studies on the subject, led to the proposal of an age and dose-dependent pattern of vitamin D supplementation efficacy, with an optimal daily dose of 700–1,000 IU and an inverted U-shaped response, along with worsening balance after the age of 70 (Sanders et al., 2010). Hiller et al. (2018) in their study included in our survey, observed that PD patients under the age of 67 improved balance with high-dose vitamin D whereas those above the age of 67 did not, confirming this age and dose-related unfavorable trend.

In summary, vitamin D supplementation in MD patients with large parts of it of older age appeared to be relatively safe and well tolerated, but further studies are essential to explore side effects in other MDs, and adverse events with high dose applications regarding balance problems, increased fracture propensity and vitamin D toxicity in old age.

### 4.3 Limitations to the study

Besides being able to present some promising results regarding efficacy and safety, our study faced several limitations. Foremost, we were confronted with substantial heterogeneity in the study designs pertaining to the study populations, doses, durations of treatment, and outcome measures. This heterogeneity precluded us from conducting a formal meta-analysis and restricted us to solely performing a qualitative analysis.

Regarding the indication of vitamin D supplement or the baseline status of patients before intervention and Vitamin D intake’s ability to raise it, Hiller et al. (2018) study stood out, as hypovitaminosis D, characterized by levels below 40 ng/mL, was the criterion for vitamin D supplementation. In contrast, the other three studies did not mandate this deficiency as a prerequisite for vitamin D treatment, and one study lacked this information altogether (Habibi et al., 2018). Importantly, while all studies, except for one, conducted 25(OH)D measurements both before and after treatment, only a singular study furnished specific numerical data (Hiller et al., 2018). Unfortunately, this omits crucial information that might help to clarify why and to what degree supplementation reached meaningful levels, as well as how it might have affected the presence or absence of beneficial outcomes.

Furthermore, there was such a scarcity of scientific evidence for the elderly population that we had to lower the age inclusion criteria. Although regrettable, this allowed us to concentrate more on the differences in vitamin D’s impact in the middle and older age groups. Hiller et al. (2018), for example, as mentioned before, observed that vitamin D improved balance in younger persons but not in older people. The authors also argued that serious research should begin around middle age, because in disorders that develop over many years or decades, supplementing should begin early and be studied for a suitable length of time.

In addition, adverse events appear to differ between age groups, despite the fact that the data from our included RCTs was insufficient to establish clear conclusions. Given the vulnerability of the aging brain (Homann, 2021), this is not surprising, but additional study is needed for confirmation.

### 4.4 Implications for research and clinical practice

#### 4.4.1 Implications for research

Discussing future research, our study highlights that there is an urgent need for extensive research on the effects of Vitamin D in MDs in middle and old age. Future studies should expand to less-studied MDs, include non-motor symptoms as outcome measure, optimize dosage, consider longer interventions, include more severe cases, employ objective outcome measures that are also sensitive to small but relevant improvements, explore diverse populations, assess safety, and perform detailed subgroup analyses. Comparative studies of middle-aged, elderly, and very old MD patients, including both clinical and molecular indicators, would be beneficial. For other conditions, such as cognitive decline or impaired mental health, vitamin D was supplemented with physical activity and other nutritional measures that proved quite beneficial in rehabilitation (Ngandu et al., 2015; Lithgow, 2019; Grzywacz and Jaron, 2020). Because there have been no RCTs on MD in this setting thus far, despite of its growing importance, this will be a crucial area to investigate. All of these initiatives will help us better understand this vulnerable population and, perhaps, provide better solutions for them.

#### 4.4.2 Implications for clinical practice

This investigation also has practical implications. It contributes to shedding more light on this generally underreported and underappreciated subject. When it comes to neuro-rehabilitation of middle-aged and older individuals with MDs, it is worth noting that also there have been RCTs investigating the effect of vitamin D together with standard pharmacological treatment and surgical based treatment there have not been investigations on vitamin D and physiotherapy interventions. However, there are promising signs that some of the effects described in the included RCTs can be particularly relevant for rehabilitation. Improving motor symptoms, balance and walking ability align well with the goals of rehabilitation in MDs patients and specifically with those of old age (Stożek et al., 2016). We strongly recommend that these outcome targets be tested in future studies in a rehabilitation environment. The poor response to high dose vitamin D on balance in older old MD patients in one of our included studies (Hiller et al., 2018) in keeping with other research of even a significant deterioration of balance in comparable age and dosage situations (Sanders et al., 2010) should lead to extra caution when these combinations are detected in patients signing up for rehabilitation programs. Currently, despite these promising indications, scientific data is insufficient to recommend vitamin D as a therapy choice in rehabilitations. Yet, considering the significant frequency of hypovitaminosis D in senior patients with MDs (Homann and Homann, 2022), as well as the demonstrated overall benefit and potential specific impact, Vitamin D management and assessment should become part of standard care for elderly patients with MDs. In this regard, replacement should be sufficient but not excessive, adhering to recommended limits (Rosen et al., 2012; Bouillon, 2017). When utilizing very high dosages, though, vitamin D and calcium levels should be closely monitored.

## 5 Conclusion

The findings of the present study suggest that the limited number of RCTs conducted thus far demonstrate potential benefits in certain populations and mostly negligible adverse effects. However, due to the fact that the eligible RCTs were confined to only two categories of MDs and the sample sizes were small, it is not possible to provide clear general recommendations regarding the use of vitamin D supplementation for the rehabilitation of middle-aged and elderly patients with MDs at this stage. Therefore, further clinical and basic science research is unequivocally necessary.

## Data availability statement

The original contributions presented in the study are included in the article/supplementary material, further inquiries can be directed to the corresponding author.

## Author contributions

CH: Project administration, Resources, Supervision, Writing – original draft, Writing – review & editing. BH: Conceptualization, Data curation, Validation, Visualization, Writing – original draft. GI: Conceptualization, Methodology, Validation, Writing – review & editing. TU-P: Conceptualization, Data curation, Formal analysis, Methodology, Writing – review & editing.
